# Analytical modelling of soil porosity and bulk density across the soil organic matter and land-use continuum

**DOI:** 10.1038/s41598-022-11099-7

**Published:** 2022-04-30

**Authors:** D. A. Robinson, A. Thomas, S. Reinsch, I. Lebron, C. J. Feeney, L. C. Maskell, C. M. Wood, F. M. Seaton, B. A. Emmett, B. J. Cosby

**Affiliations:** 1 UK Centre for Ecology & Hydrology, Environment Centre Wales, Bangor, UK; 2 UK Centre for Ecology & Hydrology, Library Ave, Bailrigg, Lancaster UK

**Keywords:** Environmental sciences, Hydrology

## Abstract

The thin layer of soil at the earth’s surface supports life, storing water and nutrients for plant uptake. These processes occur in the soil pore space, often half the soil volume, but our understanding of how this volume responds to environmental change is poor. Convention, has been to predict soil porosity, or its reciprocal bulk density (BD), from soil texture using pedotransfer functions (PTFs). A texture based approach, invariant to environmental change, prevents feedback from land use or climate change to soil porosity. Moreover, PTFs are often limited to mineral soils with < 20% soil organic matter (SOM) content. Here, we develop an analytical model to predict soil porosity, or BD, as a function of SOM. We test it on two comprehensive, methodologically consistent, temperate national-scale topsoil data sets (0–15 cm) (Wales, n = 1385; Great Britain, n = 2570). The purpose of the approach is to generate an analytical function suitable for predicting soil porosity change with SOM content, while providing insight into the main grain-scale factors determining the porosity emergence. The newly developed function covering the entire SOM gradient allows for impacts of land use, management or climate change to feedback on soil porosity or bulk density through decadal dynamic changes in SOM.

## Introduction

Climate, land use and management are important drivers of change within the earth system, and soils underpin hydrological, ecological and biogeochemical cycling within that system, and mitigate change. The magnitude, size distribution and connectivity of the soil porosity controls the flux of water and gas through soils^1^, and microbial ecosystems^2^, thus linking biogeochemical and hydrological cycling. Conversely, the bulk density (BD), which describes the mass of soil solids in a given volume, is required to determine stocks of carbon and nutrients in soil^3^; soil carbon stock is used as an indicator by the United Nations SDG 15.3.1 for assessing land degradation for example^4^. Changes to soil porosity due to compaction are one of the internationally recognized soil threats impacting plant growth and biogeochemical cycling^5^. As such, soil porosity or its reciprocal BD, are fundamental parameters in land surface, hydrological and ecological models. Most models treat porosity as a constant that results from the arrangement of sand, silt and clay particles. However, there is growing recognition in the dynamic nature of soil structure^6^, including porosity and whether it might respond to climate or land use change^7^. This leads to competing views of soils expressed in the following two hypotheses related to soil porosity dynamics:

### H1

If soil porosity or BD depends mostly on soil texture, then soil pore space evolution will be relatively ‘static’ and invariant to drivers of environmental change.

Alternatively,

### H2

If soil porosity or BD depends more on material composition and mixing, (i.e. the amount of soil organic and mineral matter) then environmental change drivers that alter the amount of SOM will result in a porosity that is ‘dynamic’ and subject to these drivers of change.

**Figure 1:**
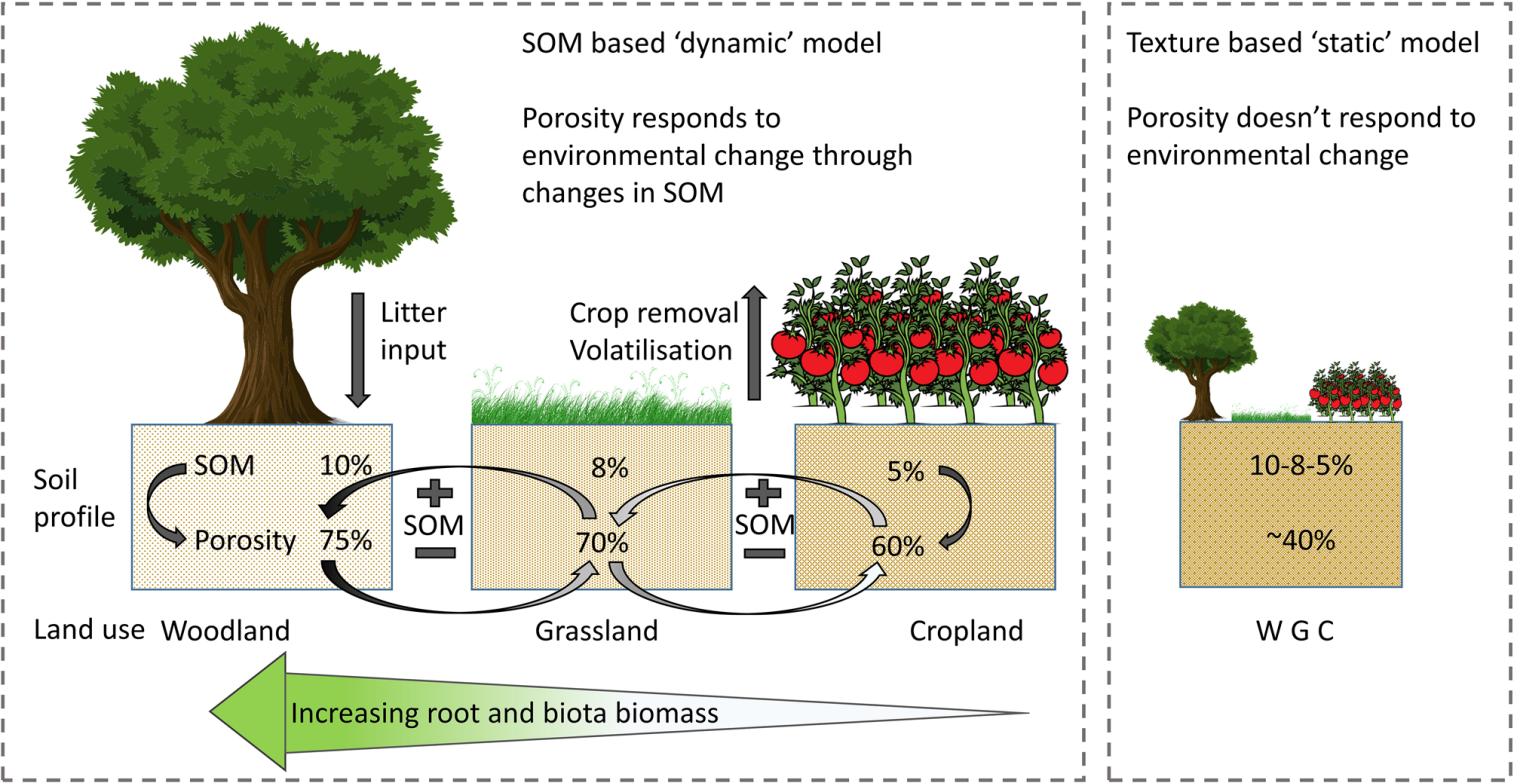
A conceptual diagram illustrating the proposed ‘dynamic’ relationship between topsoil organic matter (SOM), soil porosity and land use change, compared to the ‘static’ texture based view for the temperate UK system. High levels of SOM in broadleaf woodland result in a high porosity, transition to neutral grassland then cropland reduces the SOM and the respective porosity^36^. One might assume that it works the other way so that transition from cropland to grassland to woodland will in turn increase the porosity. While this is proposed for land use change, we would expect a similar change in soil porosity with SOM regardless of whether the change in SOM is caused by land use, management or climate change. The static view assumes no change in porosity with changing land use; *W* Woodland, *G* Grassland, *C* Cropland. Figure created by first author using Microsoft PowerPoint Version 16.0.4266.1001. https://www.microsoft.com/en-us/microsoft-365/powerpoint.

If H1 were true, the implication is that soil porosity is a relatively static property that can be treated as a constant. Whereas, if H2 were correct, then soil porosity is a dynamic variable, dependent on the rate of change of SOM. It will depend on the relationship with vegetation and carbon inputs, and carbon losses leading to feedbacks that change the porosity and associated processes such as soil hydraulic function, especially the prediction of time to ponding and runoff generation. Figure 1 attempts to demonstrate these concepts for the UK context with soil porosity giving feedback to changes in SOM. We expect any environmental driver that affects SOM concentration to potentially alter the porosity of the soil.

Compelling new research found that macroporosity in soils across the USA varied with climate^7^, something that texture-based pedotransfer functions (PTFs) would not predict. Modelling indicated that changes to soil porosity could alter soil hydraulic conductivity at soil water saturation by − 55% to + 34% by the end of the century, based on predicted changes to rainfall^7^. The mechanisms for the climate-induced variability in soil macroporosity are unknown^7^. This finding has led researchers to explore the role of soil structure on hydrological functioning using land surface models^6^. They found that, ‘soil structure significantly modified infiltration-runoff partitioning and recharge in wet and vegetated regions where more infiltration and less runoff occur, affecting deep drainage’. Nonetheless, PTFs are likely to persist in many modelling contexts; therefore, improved dynamic PTFs could yield significant benefits for land-surface models, improving representation of feedback effects. The need to determine soil carbon stocks and model hydraulic function provoke a sense of urgency to gain a fundamental physical understanding of what controls soil porosity and BD, and how they will respond to environmental change.

The material composition, (i.e. mineral and organic matter) and the microscale characteristics of soil particles, (i.e. shape and size distribution), when acted upon by the forces of nature, arrange themselves in a way that leaves space in between, filled by air or water. The resulting soil mixtures are characterized by two fundamental emergent bulk soil properties, the porosity and its reciprocal the BD, both of which are scalars. Study of soil porosity emergence and change therefore demands a stereoscopic view, both macroscopic and microscopic^8^.

**Figure 2:**
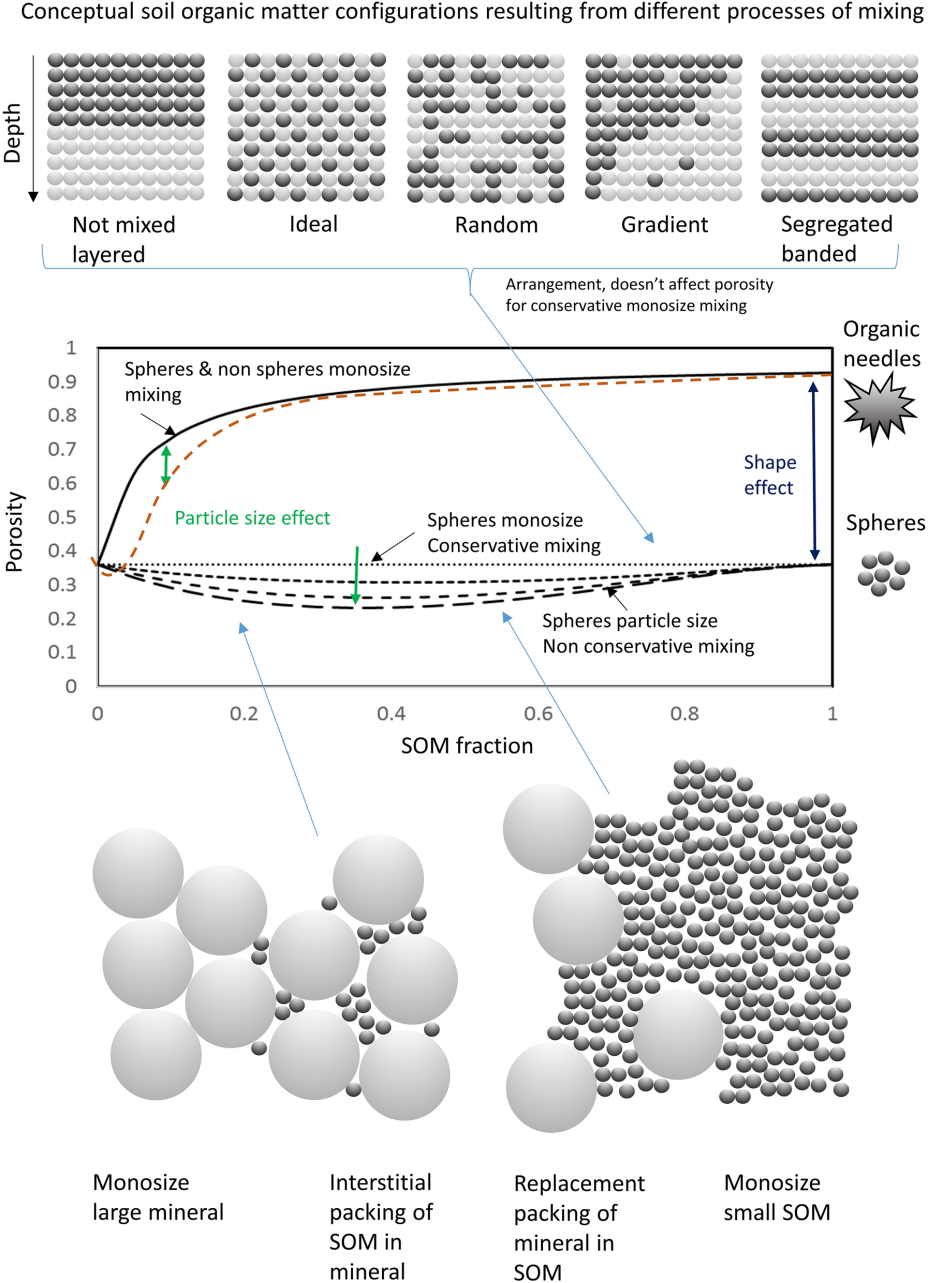
A conceptual diagram illustrating the expected response of porosity to different forms of mixing of mineral and organic matter accounting for the effects of particle shape and size distribution. The solid black line represents Eq. S18. Particle size distribution for mono-size spheres, then increasingly small spheres packed in large are shown by the dashed lines (size ratios = 0, 2.5. 5, 10), using the equations in Ref.^51^. The particles on the right side of the graph indicate the change from spherical particles to very non-spherical fibrous particles, the shape effect. The dashed orange line indicates our anticipated effect of particle size in combination with shape. Figure created by first author using Microsoft PowerPoint Version 16.0.4266.1001. https://www.microsoft.com/en-us/microsoft-365/powerpoint.

Figure 2 is a conceptual diagram of the soil grain scale. We use it to illustrate some of the important components of particle mixing and the effect on the porosity response as SOM is mixed with mineral material. At the top, five pictures of monosize spheres are illustrated that all have the same porosity but different arrangements. Recent studies focused on SOM are developing an emerging paradigm for carbon stability in soils that not only depends on chemical characteristics, but also the physical location of carbon in the soil^9^. New models of the soil system will therefore require a better description of the interplay between soil constituents, the soil mineral matrix and SOM, and how they are mixed. The different mixture ratios of minerals and SOM are examples of conservative mixing and result in a straight line in the conceptual graph as the swapping of SOM for mineral does not influence the porosity; it does affect the BD due to the difference in mass. Clearly, however, different arrangements of SOM can result from different processes. We might consider layered SOM equivalent to the formation of peaty topsoils, whereas ideal or random mixing may result from practices such as tillage that physically change the soil matrix. Naturally, we might expect gradients to form in the soil profile due to physical processes such as straining of particulate SOM from litter deposited at the soil surface or mixing due to earthworm activity, while segregation is a process that results from the flows of granular media and is more common in sediments moved by wind or water.

Particle shape can dramatically alter the porosity of a granular material. This is often modelled using spheroids as they can be dealt with relatively easily analytically. Jones and Friedman^10^ used the equation of Cumberland and Crawford^11^ to predict porosity based on changes to shape measured by altering the aspect ratio of the constituent particles. Differences in particle size can result in different forms of packing as illustrated by the spheres at the bottom of Fig. 2. In this case small particles (SOM) fit in between the larger mineral particles in binary mixing^12^. As the smaller particles fill the space between the larger particles, the interstices fill and the bulk porosity reduces. At a critical value, when all the interstices are full, the large particles are embedded in a matrix of fine particles and replace pore space until the fine particles become monosize and the original porosity is obtained. On the graph, this process is illustrated by the dashed gray line and is an example of non-conservative mixing. This does impact the porosity, reducing it as the fine particles fill the interstices of the larger ones. In studies of granular media this has been simulated for polydisperse hard sphere packings^12^. The porosity or BD depends on the size ratio of the small and large spheres and their respective quantities. Minimum soil porosity is achieved when ~ 20% small particles are mixed in large spheres with porosities of ~ 0.34 (2:1) to ~ 0.18 (10:1) and ~ 0.125 (∞). These values correspond well with measurements of environmental media (e.g.^13–15^ with porosities changing between ~ 0.15 and 0.40 m^3^ m^−3^ depending on which sizes are mixed and in what ratio. Important work has looked at the role of particle size distribution in determining soil porosity^16^, however, in soils that cover a continuum from mineral to organic, we believe SOM and particle shape are underrepresented as the characteristics that span the mineral to organic continuum explaining the form of the porosity data.

In soils, we expect all of these characteristics and processes to co-exist with dominance determined by environmental and management factors. In the case of temperate northern soils where mineral materials and SOM are mixed, our expectation is that particle shape effects, especially when considering the fibrous peat end member, will be the dominant effect on the porosity of the mixture. Hence, we focus our modelling on capturing this characteristic, which we expect to result in an upper bound for soil porosity, whilst acknowledging that particle size effects will likely cause a deviation below this model.

## Results

### Porosity and bulk density modelling

Taking a macroscopic approach for the modelling and starting with BD; ρ_b_ (g/cm^−3^) is defined as the total mass of solids (M_T_) (g) divided by the total volume (V_T_) (cm^−3^), ρ_b_ = M_T_/V_T_ where the BD is related to the porosity through the particle density (ρ_s_) of the constituents, φ = 1 − (ρ_b_/ρ_s_). Stewart et al.^17^ and Adams^18^ proposed empirical equations equivalent to (Eq. (1)) which we derive physically in the supplementary material (SI Sect. 2.0) using a conservative physical mixing model approach based on mineral and organic constituents:

1 $${\rho }_{b}=\frac{1}{\frac{SOM}{{\rho }_{bOM}}+\frac{1-SOM}{{\rho }_{bM}}}.$$

The same form of Eq. (1) is valid for determining the soil particle density^18,19^ (Eq. S14). Given that φ = 1 − (ρ_b_/ρ_s_) the porosity can be determined:

2 $$\varphi = 1 - \left[ {\left[ {\frac{SOM}{{\rho_{sOM} }} + \frac{1 - SOM}{{\rho_{sM} }}} \right] \div \left[ {\frac{SOM}{{\rho_{bOM} }} + \frac{1 - SOM}{{\rho_{bM} }}} \right]} \right],$$

where ρ_sOM_ and ρ_sM_ are the intrinsic particle densities of mineral and organic material. The resulting model requires the values for the SOM, ‘pure’ BD ρ_OM_, ρ_M_ and particle density ρ_sOM_, ρ_sM._ According to Ruehlmann and Körschens^20^ the mean particle density of the soil (ρ_soil_) can be determined using the particle densities of the SOM and mineral fractions (Eq. S14). They found for the mineral fraction that, ρ_clay_ = 2.76 g cm^−3^; ρ_silt_ = 2.69; ρ_sand_ = 2.66; and proposed for the SOM fraction that a dense (ρ_SOMhd_ = 1.43 g cm^−3^) and light (ρ_SOMld_ = 1.27) organic fraction could be identified. Where they equate the ρ_SOMld_ to the microbial biomass and ρ_SOMhd_ to the more dense humified material, for example that occurs in peats and organo-mineral soils. In recent work, Ruehlmann^19^ collected a comprehensive data set for particle densities of soils for the full range of SOM. We use this data to determine the particle density for our end members in mineral (ρ_sM_ 2.7 g cm^−3^) and organic (ρ_sOM_ 1.4 g cm^−3^) soils (Fig. S1).

Adopting a microscale, or grain scale approach, we are able to exploit recent advances in soft matter physics to gain physical insight into the emergent macro-scale properties^21^. Studies in soft matter help provide vital understanding into the behavior of granular media. From such studies we know that grain shape, particle size distribution (PSD), repulsion forces and particle friction (*µ*) are all factors that contribute to the way in which granular media pack^22,23^. Lattices of spheres have been a source of practical and theoretical interest for millennia. The Kepler conjecture, is perhaps one of the best-known mathematical theorems about 3D mono-size sphere packing. It states that, ‘no packing of congruent balls in Euclidean three-space has density greater than that of the face-centered cubic packing’^24^. The packing density (*η* = V_sphere_/V_unit cell_) or porosity (φ = 1 − *η*) of lattices of spheres are well known and can be determined mathematically, although the formal proof was only published recently^25^. They include face centred cubic (π/(3√2) ≈ 0.740 m^3^ m^−3^); body centred cubic ((π√3)/8 ≈ 0.680 m^3^ m^−3^) and simple cubic (π/6 ≈ 0.524 m^3^ m^−3^) for example, with porosity ranging between ~ 0.26 and 0.47 m^3^ m^−3^. However, packings of granular media are generally disordered and much more challenging to describe.

Jammed packing’s are used to describe disordered materials, with the ‘maximally jammed random state’ describing the lower bound porosity attainable for mono-size spheres (~ 0.36 m^3^ m^−3^)^22^. Studies of the packing of disordered materials have made substantial progress using computers in soft matter physics, and are largely applicable to the problem of soils, or sediments, which are disordered materials. The problem of determining the porosity of disordered spheres has proved a problem of substantial interest^21^. Song et al.^26^ presented a mathematical solution to the problem and composed a phase diagram for the packing of disordered hard spheres. The theory begins by determining the relationship between the free volume of the particle (*W*) and the geometrical coordination number (*z*):

3 $$W\left(z\right)=\frac{2\sqrt{3}}{z}{\text{V}_{g}},$$

where V_g_ is the volume of the grains. It shows that *W*, is inversely related to *z*. From this starting point they derived an equation of state relating the packing density (*η* = 1 − φ) to coordination number (*z*):

4 $$\eta =\frac{z}{z+2\sqrt{3}}.$$

They found that for the ground state or maximally jammed random state *η* = 0.634 for frictionless particles (*µ* = 0) with z = 6, whereas for the maximally jammed loose state *η* = 0.591 as *µ* → ∞ with z = 4. The relationship between packing density and particle friction is presented in Ref.^27^ with a non-linear relationship. These values correspond well with environmental granular media like sandy soils and sediments (φ ≈ 0.4).

Particle shape orientation and size distribution are also important factors that change the way particles pack^22^. In this work we incorporate an empirical geometric factor related to shape and acknowledge in our conceptual framework (Fig. S1) that a complimentary model will exist that incorporates particle size, especially where small particles fill the interstices of the large ones. The grain scale approach provides important insight showing that the porosity or BD will depend on characteristics such as the coordination number with other particles, particle shape, size distribution, surface friction and repulsive forces. Moreover, if we combine the macroscale approach (Supplementary Eq. S13) with the microscale approach (Eq. (4)) we can obtain the following equations for mono-size spheres:

5 $$\varphi =\frac{2\sqrt{3}}{z(\mu )+2\sqrt{3}},$$

6 $${\rho }_{b}=\frac{{\rho }_{s}z(\mu )}{z(\mu )+2\sqrt{3}}.$$

Further assumptions can be made, for example that 2√3 can be multiplied by an empirical adjustable geometrical factor $2\sqrt{3}\times GF$. By exchanging Eq. (6) into Eq. (1) we obtain new approximations for soil porosity or BD (Eqs. (7), (8)). Where, GF is an empirical geometrical factor > 1 and *z* is an effective coordination number which, has some power law dependence on the particles friction (*µ*)^27^; other symbols are as previously defined:

7 $$\varphi = 1 - \left[ {\left[ {\frac{SOM}{{\rho_{sOM} }} + \frac{1 - SOM}{{\rho_{sM} }}} \right] \div \left[ {\frac{SOM}{{\frac{{\rho_{sOM} z\left( {\mu_{OM} } \right)}}{{z\left( {\mu_{OM} } \right) + \left( {2\sqrt 3 \times GF_{OM} } \right)}}}} + \frac{1 - SOM}{{\frac{{\rho_{sM} z\left( {\mu_{M} } \right)}}{{z\left( {\mu_{M} } \right) + \left( {2\sqrt 3 \times GF_{M} } \right)}}}}} \right]} \right],$$

8 $${\rho }_{b}=\frac{{\rho }_{sM}{z({\mu }_{M})\rho }_{sOM}z({\mu }_{OM})}{SOM\times {\rho }_{sM} z\left({\mu }_{M}\right)\left(z\left({\mu }_{OM}\right)+(2\sqrt{3}\times {GF}_{OM})\right)+{\rho }_{sOM}z({\mu }_{OM})(-SOM+1)(z\left({\mu }_{M}\right)\times (2\sqrt{3}\times {GF}_{M}))}.$$

The result is a set of computationally simple analytical equations that describe the porosity or BD of granular materials with monosize particles that can be adjusted for grain geometry through an empirical geometric factor. Strictly, the models do not include particle size effects that would result in non-conservative mixing and we therefore expect the models to form an upper bound for the physical characteristics of the soil. The contribution of repulsion forces is neglected for temperate, coarse to loamy textured soils, given the wetting and drying cycles resulting in cohesion from suction forces, and organo-mineral particle stabilization. Moreover, data shows that low activity clays, or those with no surface charge such as talc^28^, produce high porosities (> 0.70 m^3^ m^−3^) showing the importance of geometry; we expect counter ions in soils will minimize the effect of electrostatic forces such that geometric factors outweigh electrostatic repulsive forces. Nor do the equations provide insight into the arrangement of the particles, as we point out in our development of conceptual theory and Fig. 2.

### National soil data

**Figure 3:**
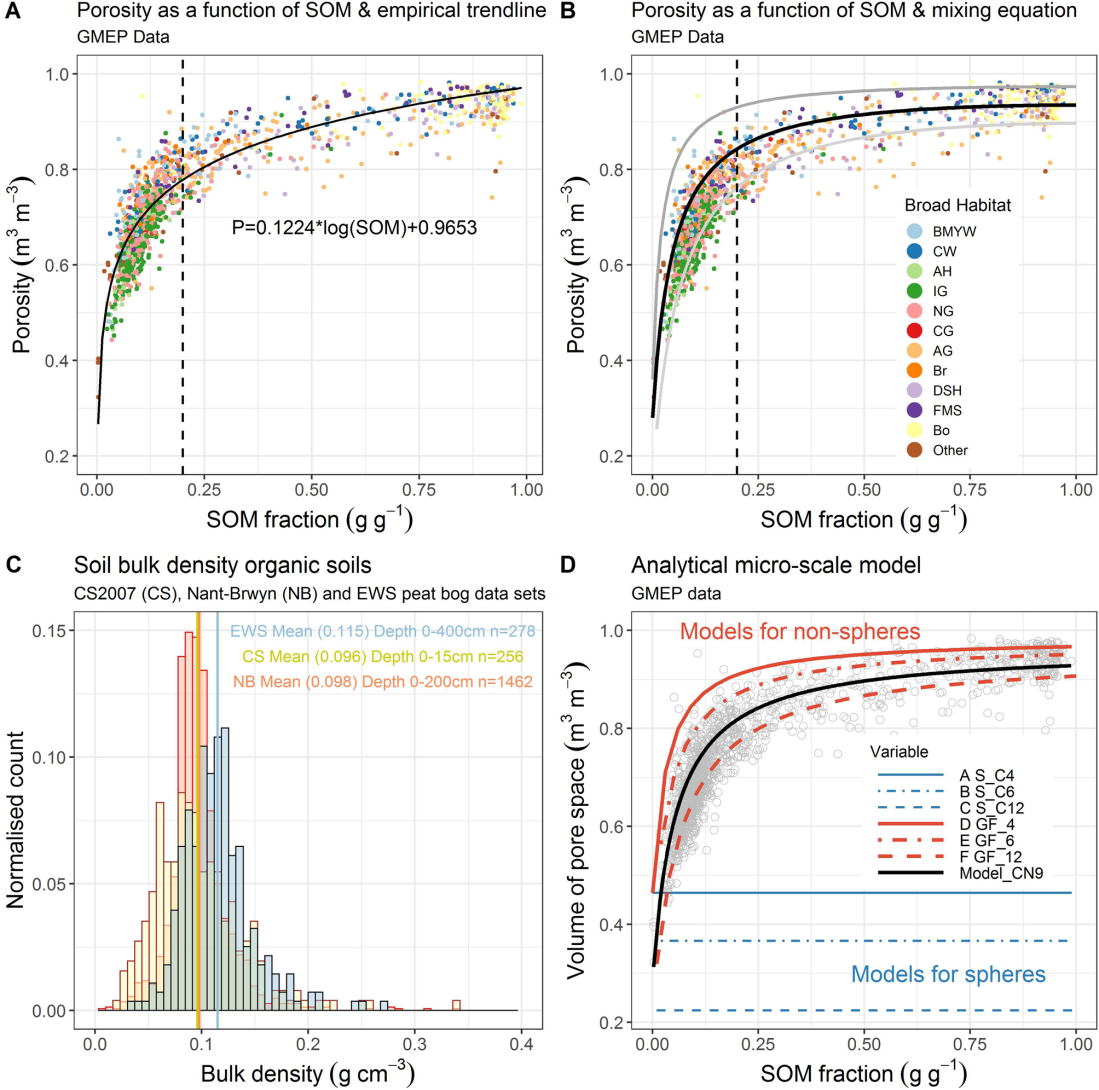
(**A**) Soil porosity as a function of soil organic matter (SOM) content from the GMEP data set^29^ n = 1385. An empirical black trendline is fitted to the data (r^2^ = 0.81 (actual vs predicted); RMSE = 0.05). The dashed line marks the transition from mineral soils (SOM < 0.2) to organic soils (SOM > 0.2). Markers are colour coded by Broad Habitat: broad-leaved woodland (BMYW) coniferous woodland (CW), arable and horticultural (AH), improved (IG), neutral (NG), calcareous (CG) and acid (AG) grassland, bracken (Br), dwarf shrub heath (DSH), fen marsh swamp (FMS) and bog (Bo). (**B**) The GMEP data set with the black line modelled using Eq. (1) assuming, intrinsic particle densities ρs_OM_ = 1.4 g cm^−3^ and ρs_M_ = 2.7 g cm^−3^ and values for the ‘pure’ SOM BD (ρ_OM_ = 0.09 g cm^−3^) and mineral BD (ρ_M_ = 1.94 g cm^−3^). The thick grey line above and the thin grey line below the black line represent approximate bounds based on porosities for mineral granular media^13,14^ and the standard deviation for the organic soil (SOM > 0.9). (**C**) Histograms of BD of organic soils from 3 data sets (EWS, UKCEH Countryside Survey and NB Migneint) described in the “Materials and methods”, “Organic soil bulk density data” section, n = 1462. (**D**) Soil porosity as a function of SOM for the GMEP data set, grey markers. The 3 blue lines are modelled with Eq. (7) assuming spheres with coordination numbers of 4, 6 and 12. The black line is modelled based on Eq. (7) assuming, intrinsic particle densities ρs_OM_ = 1.4 g cm^−3^ and ρs_M_ = 2.7 g cm^−3^ and values for $2\sqrt{3}\times {GF}_{OM}$ = 117; z_OM_ = 9 determined by minimizing the sum of squares error; *µ* is discounted. The red lines represent the same model parameters with the coordination number altered to 4, 6 and 12.

Figure 3 shows a strong dependence of soil porosity on SOM using a national data set. The data show a clear non-linear change in soil porosity with SOM, modelled with an empirical logarithmic function to show the trend (r^2^ = 0.81 (actual vs predicted); RMSE = 0.05) (Fig. 3A). Comprehensive national data sets that contain the full range of SOM and BD across land uses are limited, and the lack of samples in soils with higher SOM has prevented previous studies from identifying this trend. The GMEP data set for Wales^29^ (Fig. 3) offers a comprehensive set of statistically robust topsoil measurements (0–15 cm including SOM and BD) from mineral to organic and for a range of broad habitats. The data clearly show a change in the porosity with the transition across land uses. Porosities are lower for arable (AH) (mean = 0.53 m^3^ m^−3^) and improved grassland soils (IG). The broad-leaved woodland (BMYW) (mean = 0.70), neutral (NG) and calcareous (CG) grassland occupy the higher porosities where mineral soils transition to organic. The dashed line represents this transition to organic soils that are occupied by unimproved acid grassland (AG), bracken (Br), coniferous woodland (CW) and dwarf shrub heath (DSH) migrating to the peats in fen march swamp (FMS) and bog (Bo) (mean = 0.91 m^3^ m^−3^).

### Soil mineral and organic matter conservative mixing model

How well does the macroscale analytical mixing approach (Eqs. (1), (2)) predict porosity? As proposed in the conceptual development Eq. (2) fits the form of the data well when applied to the empirical data in Fig. 3B, anchored by its end members. The black line through the data (Eq. (2)) relies on the following values for the end member intrinsic particle densities ρ_sOM_ = 1.4 g cm^−3^ and ρ_sM_ = 2.7 g cm^−3^. These are determined from Ruehlmann^19^ as previously described (Fig S1), and values for the ‘pure’ SOM BD (ρ_bOM_ = 0.10 g cm^−3^ ≈ φ = 0.93 m^3^ m^−3^) and mineral BD (ρ_bM_ = 1.95 g cm^−3^ ≈ φ = 0.28 m^3^ m^−3^). The value of ρ_bOM_ is representative of independent values in Fig. 3C. This approach and value are consistent with a number of national data sets presented in Fig. 3C where ~ 2000 measurements in three independent data sets indicate the same approximate value for the BD of mostly uncompact organic soils. The Fig. 3B end points for the porosity of the mineral materials (upper φ = 0.36; lower φ = 0.15 m^3^ m^−3^) are taken from Ref.^14^ for monosize grains (upper) and Ref.^13^ for the extreme case of clay compressed in sand at a confining pressure of 30 MPa (lower), the value of 0.28 comes from the former for a binary sand mixture. The standard deviation is used to determine the SOM porosities (0.97 and 0.90 m^3^ m^−3^). Based on these data and assumptions the model describes the data reasonably well giving a slightly lower fit as expected as it forms an upper bound and is not fitted to the data (r^2^ = 0.70; RMSE = 0.06). The values are not known a priori and a range of BD values have been presented in the literature, but all from small or incomplete data sets. The BD of organic soils seems to have attracted the least attention in the literature. We consider organic soil and peat independently, with the assumption that peat is likely to represent the organic end member. Perie and Ouimet’s^30^ organic forest soils resulted in a value of ρ_OM_ of 0.111 g cm^−3^. Whereas, peat soils tend to lie in the range 0.1–0.2 g cm^−3^ from around the world depending on the degree of humification and compaction^31,32^. Hence, assuming peat is the end member, we find an average value of ρ_OM_ of ~ 0.1 g cm^-3^ seems appropriate based on the range of evidence for uncompact peat. These results clearly show the dependence of porosity or BD on SOM. Soil formation, environmental, management or degradation factors that lead to changes in SOM will inevitably lead to changes in porosity or BD. Given changes to SOM levels in soils are observed on decadal time scales, we should expect changes in hydraulic function on a similar time scale.

### Soil mineral and organic matter mixing model including grain scale

**Figure 4:**
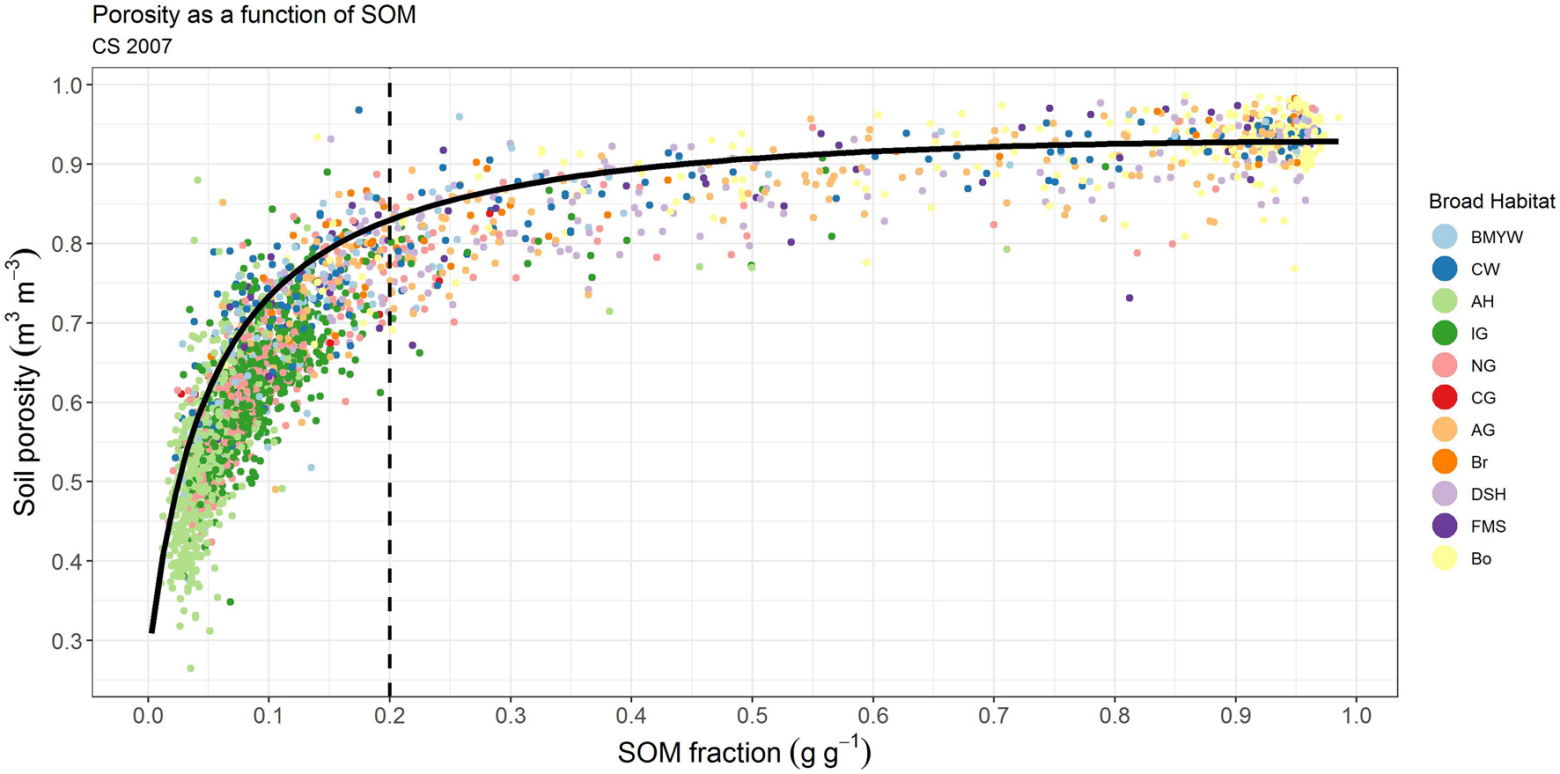
Soil porosity as a function of soil organic matter (SOM) content from the independent UKCEH Countryside Survey 2007 data set^36^ n = 2570. The dashed line marks the transition from mineral soils (SOM < 0.2) to organic soils (SOM > 0.2). Markers are color coded by Broad Habitat, broad-leaved woodland (BMYW) coniferous woodland (CW), arable and horticultural (AH), improved (IG), neutral (NG), calcareous (CG) and acid (AG) grassland, bracken (Br), dwarf shrub heath (DSH), fen marsh swamp (FMS) and bog (Bo). The black line is the model (Eq. (7)) with CN = 9 and assuming intrinsic particle densities ρs_OM_ = 1.4 g cm^−3^ and ρs_M_ = 2.7 g cm^−3^ and values for $2\sqrt{3}\times {GF}_{OM}$ = 117; z_OM_ = 9 as used previously on the GMEP data; *µ* is discounted.

The new porosity (Eq. (7)) and BD (Eq. (8)) models provide an opportunity to explore the data in ways others. In Fig. 3D we apply Eq. (7) to predict porosity (see Fig. S2 for BD), initially with the assumption that all particles are spheres and either mineral or organic (Fig. 3D, straight pale blue lines, A–C). According to the theory of Ref.^26^ *z*, should lie between 4 (Fig. 3D, A) and 6 (Fig. 3D, B) for a maximally jammed random state for monosize spheres, while 12 (Fig. 3D, C) is the lower bound for monosize spheres (Face Centred Cubic). Clearly from Fig. 3D the mineral materials with SOM = 0 are approximated by the model, but the organic materials SOM = 1 are not. This is unsurprising as the fibrous nature of peat is hardly spherical. In the next step we maintained the sphere geometry for the mineral component and determined the values for *z* and $2\sqrt{3}\times {GF}_{OM}$ by fitting the analytical model (Eq. (7)) to the empirical model (Eq. (2), Fig. 3B), assuming the same values for end member intrinsic particle densities ρ_sOM_ = 1.4 g cm^−3^ and ρ_sM_ = 2.7 g cm^−3^. Therefore, the values of *z* and $2\sqrt{3}\times {GF}_{OM}$ are dependent on the BD of the endpoints and not the data as a whole. The values obtained were 9 for an ‘effective coordination number’ (CN) and 117 for the SOM geometric factor ($2\sqrt{3}\times {GF}_{OM}$), the r^2^ of the actual vs predicted (fit being r^2^ = 0.73; RMSE = 0.06). A value of 9 is at the higher end and may be due to mixing of different shapes, or missing physics, such as particle size distribution and repulsion forces^23^. We then replaced $2\sqrt{3}\times {GF}_{OM}$ with the new empirical value of 117 ($2\sqrt{3}\times 33.77\dots$) and plotted the model predictions for the coordination numbers (CN) of 4 (D), 6 (E) and 12 (F) in Fig. 3D. The modelled data gives a good description for an upper bound to the GMEP data (Fig. 3D). A further independent test was performed by comparing the model (Eq. (7)) with the UKCEH Countryside Survey data which has a much broader spatial coverage and number of habitat locations (n = 2570). The model, without fitting to the data provides a clear, expected, upper bound for the data (Fig. 4).

What do we learn from this new model? The key findings are that the mixing model (Eq. (7)) is an appropriate way to describe an upper bound for the non-linear data (Figs. 3D, 4), but that in order to capture the data fully a non-spherical geometric factor must be applied for the SOM (Fig. 3D, D,GF_4 to F,GF_12). Assuming the model in Fig. 3D is a reasonable approximation of the upper bound, deviation below this could be for a number of physical reasons. Increasing contact resulting from mixing particles of different shapes could partly account for this, as we demonstrate increasing CN to 12. Moreover, particle size, not accounted for in this model, but shown conceptually in Fig. 2 may also account for this reduction in porosity for a given SOM level; while particle arrangement, such as bridging and clustering brought about by friction for example or soil compaction may also play roles. Our results support the observation^33^ that using the empirical form of the model (Eqs. (1) or (2)) fitted to data will result in a good r^2^ but with a high RMSE. This high RMSE eludes to the missing physical components not contained in the model, e.g. particle size and compaction for example. Unravelling the complexity to explain the residuals is challenging, but important from both a theoretical point of view, and a practical point of view for prediction. We would also expect that broadening the data set to soils beyond the temperate region, into dryland or low organic matter soils would increase the dominance of particle size for example, as found by others for low SOM soils^16,33^.

### Exploration of the model residuals

**Figure 5:**
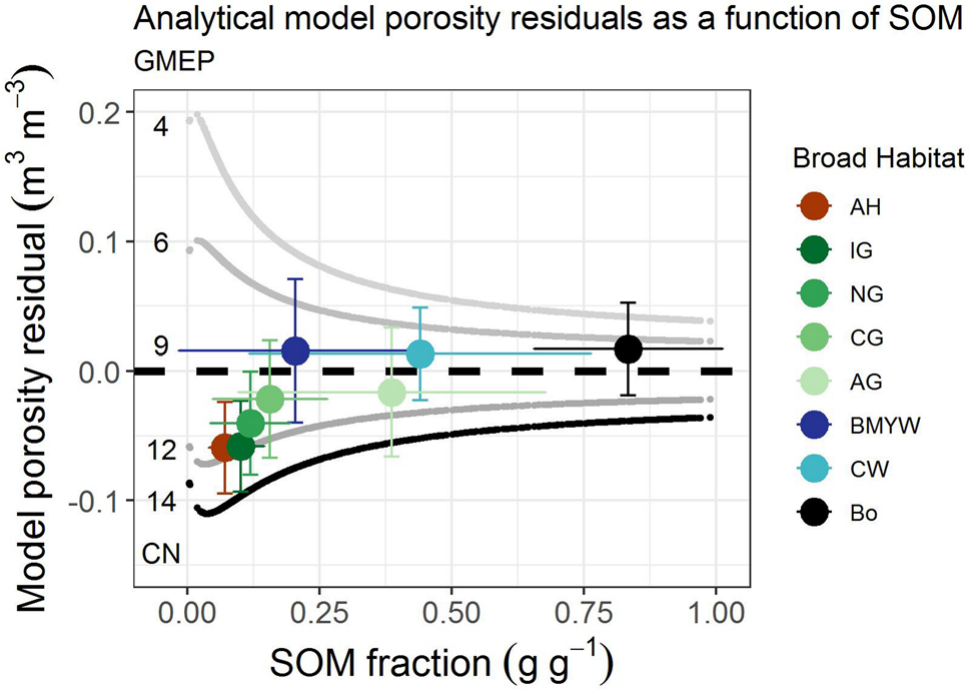
Sensitivity plot for the porosity residuals for the main broad habitats from the model (Eq. (7), black line plotted in Fig. 3D, CN = coordination number) assuming, intrinsic particle densities ρs_OM_ = 1.4 g cm^−3^ and ρs_M_ = 2.7 g cm^−3^ and values for the ‘pure’ SOM BD (ρ_OM_ = 0.09 g cm^−3^) and mineral BD (ρ_M_ = 1.94 g cm^−3^) vs SOM. The upper and lower curved lines are the approximate bounds. The solid circle markers represent the mean response by habitat type (Fig. 4 caption) with standard deviation error bars. The highly managed arable (red) and improved grassland (dark green) show the largest deviation from the model.

**Figure 6:**
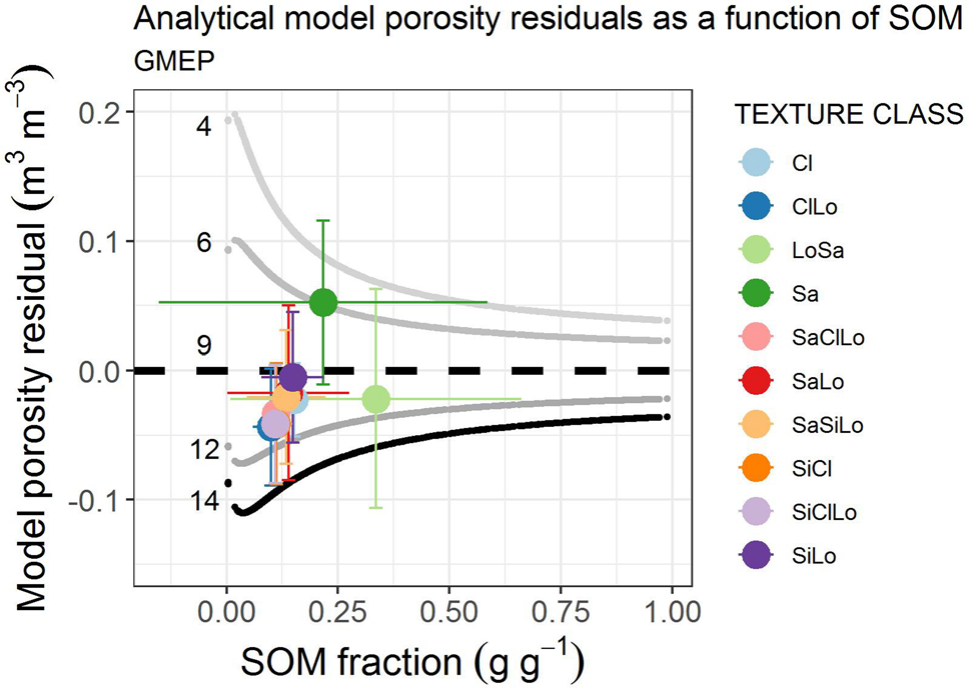
Residuals plotted as a function of SOM for the respective soil texture groups. Markers are color coded by texture class: clay (Cl), clay loam (ClLo), loamy sand (LoSa), sand (Sa), sandy clay loam (SaClLo), sandy loam (SaLo), sandy silty loam (SaSiLo), silty clay (SiCl), silty clay loam (SiClLo), silty loam (SiLo). Median CN values for each texture class shown in Supplementary Table S1. The residuals show a clear particle size effect in the expected direction. The coarse sandy soils having the lowest coordination number and the fine grained clay soils with a broad particle size distribution having the highest CN. This indicates that CN contains both the shape effect and the secondary particle size effect.

Figure 5 explores how soil porosity in different habitats diverges from the analytical model. We use Eq. (7) (Fig. 3D) to explore the residuals as a function of SOM, colored by broad habitat (BH). The porosity residuals plot generated is bounded by the analytical model predictions for different coordination numbers (Eq. (7)). The abscissa corresponds with CN = 9, and the upper grey lines CN = 6 and 4, while the lower darker lines are CN = 12 and 14. The response for each BH and their respective standard deviations are shown by the error bars (see Fig. S3 for the raw scattered data). We would expect that if there is no bias that the soils would be randomly clustered around the zero line (CN = 9). Those exhibiting higher porosity will sit above the line and those with lower will sit below the line. The data shows a clear deviation from the zero line for soils with SOM < 0.2 g g^−1^ (Fig. 5, Suppl. Fig. S3). These data points represent the managed habitats on mineral soils. The most likely explanation for this is the lack of representation of particle size in the model for mineral soils. The indication that these soils have CN of 12 or greater contact points is only physically feasible if the particle size is greater than monosize. The monosize limit is CN = 12 and only for ordered structures, which is not the case for soils. By plotting the particle size classes on the residual plot (Fig. 6) there does appear to be some of this relationship evident. CN can be determined from the bulk density and SOM by rearranging Eq. (8) to make CN the subject. We used these to determine a median value for CN for each texture class (Supplementary Table S1). The results appear reasonable with sand having the lowest CN values and clay loams and silty clay loams, which have broad PSD, having the highest. The coordination number for sands was 7.3, which compares favorably to measured CN values for natural sands ranging from 6 to 8^34^. Moreover, the CN value increases quickly as sphericity reduces^35^; in natural beach sand rising from ~ 6 to close to 20 for angular sands. We inserted the appropriate median value of CN into the model for each particle size class. By doing this the r^2^ and RMSE increased from r^2^ = 0.73 and RMSE = 0.06 for CN = 9 to r^2^ = 0.82 and RMSE 0.05 m^3^ m^−3^. This is an improvement but highlights that the porosity is a complex interplay between grain shape and size distribution. The dominance of particle shape as a factor over particle size distribution is consistent with other research on physical response, where particle size is a secondary factor, compared to mixing and shape for example, for the electrical properties of porous media like soils^14^. Moreover, other factors such as rooting, bioturbation and compaction are not accounted for but the high level of variance explained indicates that the major contributing factors in these soils are SOM, particle shape and particle size. These three factors appear to be the dominant characteristics of the soil that determine the emergent nature of soil porosity. However, the relative order of importance will depend on the dominant climatic and soil characteristics. For instance, in drylands with very low SOM particle size and shape are likely to be dominant.

## Discussion

We can see that the porosity or its reciprocal BD largely depends on SOM, particle shape and to a lesser extent particle size in these soils, for this range of SOM. From the form of the model we can also see that soil porosity is much less sensitive to SOM in organic soils (SOM > 0.2 g g^−1^) and much more sensitive to SOM in mineral soils (SOM < 0.2 g g^−1^). Reduction of SOM from 0.1 to 0.05 g g^−1^, the difference between an improved grassland and an arable soil^36^, results in a reduction in porosity of ~ 0.15 m^3^ m^−3^. This indicates a much lower soil storage capacity. The relationship established shows the importance of the mixing of mineral and organic material. This supports the importance of the link between vegetation, carbon inputs to soil and the emergent porosity of the soil. The vegetation inputs and carbon cycling will thus provide an important feedback to soil porosity, thereby identifying one potential mechanism for the observed change in porosity with land use change or climate^7^.

Based on the SOM contents in the Countryside Survey data set^36^ (SOM = 1.81 × SOC) we might expect porosity to increase from ~ 0.60 for arable to ~ 0.7 for improved grass to ~ 0.75 m^3^ m^−3^ for broadleaved woodland. In the case of the GMEP data set for Wales the results are broader with a soil porosity of ~ 0.55 expected for improved land (arable & improved grass) to ~ 0.8 m^3^ m^−3^ for woodland^37^. Contrast this to texture based PTF predictions like the one in Hydrus 1D^38^ which predicts a soil porosity of ~ 0.44 for improved land and ~ 0.41 m^3^ m^−3^ for woodland based on the median texture for the habitats in the GMEP data^37^. This indicates how land use change and alterations in SOM could play a major part in the emergent porosity of soils. This feedback, to our knowledge remains unaccounted for in land surface models with unknown impacts on biogeochemical and hydrological cycling. This linkage is an often-overlooked aspect of determining soil porosity, and is absent, when porosity is predicted based simply on texture through PTFs. While inputs for bulk density^38^ and soil carbon have now been incorporated^39^, these data can be limited in national data sets, especially bulk density, or the PTF development tends to be dominated by, or limited to, mineral soils with SOM concentrations < 0.2 g g^−1^. This is a limitation for both hydraulic land surface modelling where we need to understand how soil porosity changes across all soils and biomes, and for carbon density prediction that requires determination of soil bulk density across soils and biomes. Based on the function presented, given SOM data is available, there is the potential to predict soil porosity or BD which may improve prediction of hydraulic properties over and above texture alone. At present, land surface models like JULES^40^ use porosity predicted using PTF’s with the limitation to mineral soils with SOM concentrations < 0.2 g g^−1^, or they include an empirical porosity value for organic soils. We contend that the use of an empirical function, or the simple form of the analytical model (Eq. (2)), avoiding multiple parameters, could substantially improve porosity prediction and its change with SOM change due to land use change for example. Such a resulting function could be implemented easily in physically based land surface, climate or ecological models with a soil component. The reason this becomes important is for determining time to soil water ponding and run-off generation^6^. Underestimating porosity may lead to premature run-off generation.

In this work we have built on early research that explored whether BD could be modelled knowing the proportion of organic and mineral soil components^17,18^. The early work was largely subsumed by a greater focus on soil hydraulic functions^41^ and linking them to soil texture, e.g.^42,43^ and the development of pedotransfer functions^44–46^ which were driven by arable agriculture. Development of computing power and statistical methods led to the development of more sophisticated algorithms to explore the relationships between soil constituents and porosity or BD^39^, but research was heavily skewed to arable soils often in drylands. Moreover, the recognition of soils as the major terrestrial carbon store has stimulated interest in determining BD to estimate carbon (and nutrient) stocks^3^. However, the application of these somewhat ‘black box’ machine learning approaches to determining BD in isolation has led to better fitting but less new insight being gained, as pointed out by Tranter^33^. Tranter et al. attempted to reconcile and compare empirical models, testing a range of models on a large Australian soil data set (n = 1896), but understanding was again limited by low SOM concentrations and the focus on mineral soils, much the same as the large (n = 2721) US data sets previously tested^45^. Their work was important, but the work here gives greater balance by including soils in temperate regions with a full spectrum of SOM levels. While we appreciate that machine learning methods will likely produce a better data fit, this simple physical model is a powerful demonstration of the insight gained for the major contributing factors of SOM, particle shape and particle size to the porosity of the soil. An important future challenge is to develop a methodology to assess porosity, and its potential change, using easily obtained data, for example from remote sensing. This would greatly advance our ability to estimate both porosity for hydraulic function and carbon stocks for climate change research.

## Conclusions

Using an analytical modelling approach, we determine a simple model for soil porosity and bulk density, based on grain-scale soil characteristics. Testing our model against large national datasets across a large range of SOM concentrations we conclude by favoring the second hypothesis posed in this work: soil porosity will respond ‘dynamically’ to changes in SOM which itself can be driven by changes in land use or climate on the time scale of SOM change. Hence, the incorporation of a dynamic mechanism in land surface hydrological or ecological models will generate feedbacks to earth system biogeochemical or hydrological cycles important for understanding land use and climate change effects on ecosystems.

## Materials and methods

### Study area

The research used two study areas in the UK. The GMEP data was collected between 2013 and 2016 from across Wales, while the UKCEH Countryside Survey 2007 data was collected from across Great Britain (England, Scotland and Wales) during 2007. All the details of all the methods used to analyze the soil samples can be found in the supporting information of the respective data sets described below. Only basic information is provided here.

### GMEP data

Topsoil samples (0–15 cm) were collected from across Wales based on a stratified random design using land class, a combination of parent material, climate and relief^47^, to stratify. N = 1385 measurements are presented in this work^29^. Soil Organic Matter (SOM) was determined using loss on ignition and bulk density was determined on oven dry fine earth (< 2 mm) samples. All methods were based on and compatible with the UKCEH Countryside Survey^48^. Histograms of the data for SOM (Fig. S4) and bulk density (Fig. S5) are presented in the Supplementary Information.

### UKCEH Countryside Survey data

Topsoil samples (0–15 cm) were collected from across Great Britain in 2007 based on a stratified random design using land class^47^, to stratify. N = 2570 measurements are presented in this work^48^. Soil Organic Matter (SOM) was determined using loss on ignition and bulk density was determined on oven dry fine earth (< 2 mm) samples. All methods were based on the UKCEH Countryside Survey as detailed in the supporting information in Ref.^48^. Histograms of the data for SOM (Fig. S6) and bulk density (Fig. S7) are presented in the Supplementary Material.

### UKCEH Countryside Survey data subset

These are topsoil data as previously described that were subset for soils with SOM > 0.9 g g^−1^ (n = 256)^48^.

### Organic soil bulk density data

Organic soil bulk density data shown in Fig. 3C was obtained from 3 independent data sets^49^.

### EWS data

“Cores were collected from five upland ombrotrophic blanket bogs selected to represent a latitudinal gradient through Great Britain. Triplicate adjacent cores were extracted in 2014 using both a box corer (to recover the surface vegetation and uppermost 1 m of peat) and a Russian-type corer (for peat deeper than 1 m). The sites were: Great Gnat’s Head on Dartmoor (DM), Migneint (Mg) in northwest Wales, Moor House (MH) in northern England, Glensaugh (G) near the north eastern Scottish coast and Forsinard Flows (F) in the far north of mainland Scotland. All cores extended down to the underlying mineral substrate, ranging in length from 95 (Glensaugh) to 417 cm (Forsinard). The new cores were carefully extruded, sliced at 10 cm intervals, air-dried for one week, manually sieved to 2 mm to remove large particles and roots, oven-dried at 60 °C to remove residual moisture and ball-milled to a fine, homogenous powder to determine SOM from loss on ignition. Bulk densities of all samples were calculated prior to milling by dividing the dry matter mass by the original volume of wet material” n = 278^49^.

### Nant Brwyn data

Data were collected from the Migneint (Mg) in northwest Wales during May and June 2011. “At each sample location the soil was sampled with a Russian auger with a flight of length 50 cm and an estimated sample volume of 622 cm^3^. Samples were collected up to depth 2 m. The samples were cut into 10-cm sections. On return to the laboratory, samples were placed in an oven to dry at 105 °C for 72 h. From these measurements the dry BD was computed for each 10-cm section. Organic carbon content was determined on material from each section by loss on ignition” (n = 1462)^50^.

### Analytical modelling

Theory development described in the Supplementary Information.

## Supplementary Information

Supplementary Information.

## Data Availability

The main datasets used in the paper are directly available from the Environmental Information Data Centre (EIDC) through the following links: Emmett, B.A.; Reynolds, B.; Chamberlain, P.M.; Rowe, E.; Spurgeon, D.; Brittain, S.A.; Frogbrook, Z.; Hughes, S.; Lawlor, A.J.; Poskitt, J.; Potter, E.; Robinson, D.A.; Scott, A.; Wood, C.M.; Woods, C. (2016). Soil physico-chemical properties 2007 [Countryside Survey]. NERC Environmental Information Data Centre. (Dataset). 10.5285/79669141-cde5-49f0-b24d-f3c6a1a52db8. Lebron, I.; Seaton, F.; Barrett, G.; Alison, J.; Burden, A.; Emmett, B.A.; Garbutt, A.; Robinson, D.A.; Williams, B.; Wood, C.M. (2020). Topsoil particle size distribution from the Glastir Monitoring and Evaluation Programme, Wales 2013–2016. NERC Environmental Information Data Centre. (Dataset). 10.5285/d6c3cc3c-a7b7-48b2-9e61-d07454639656. Robinson, D.A.; Astbury, S.; Barrett, G.; Burden, A.; Carter, H.; Emmett, B.A.; Garbutt, A.; Giampieri, C.; Hall, J.; Henrys, P.; Hughes, S.; Hunt, A.; Jarvis, S.; Jones, D.L.; Keenan, P.; Lebron, I.; Nunez, D.; Owen, A.; Patel, M.; Pereira, M.G.; Seaton, F.; Sharps, K.; Tanna, B.; Thompson, N.; Williams, B.; Wood, C.M. (2019). Topsoil physico-chemical properties from the Glastir Monitoring and Evaluation Programme, Wales 2013–2016. NERC Environmental Information Data Centre. (Dataset). 10.5285/0fa51dc6-1537-4ad6-9d06-e476c137ed09. Toberman, H.; Tipping, E.; NERC RadioCarbon Facility; Somerville, C.; Helliwell, R.; Carter, H.; Keenan, P.; Pereira, M.G.; Patel, M.; Tanna, B.; Thompson, N.; Bryant, C.; Elliott, F.; Gulliver, P. (2016). Peat survey in England, Scotland and Wales carried out during 2014 [LTLS]. NERC Environmental Information Data Centre. (Dataset). 10.5285/9305b068-f417-4659-9966-d9456f22c331.
