# Supplementary material for: Analytical modelling of soil porosity and bulk density across the soil organic matter and land-use continuum
Source: Sci Rep. 2022 Apr 30;12:7085. doi: 10.1038/s41598-022-11099-7 (PMC9056517; doi:10.1038/s41598-022-11099-7)
Supplement: Supplementary file 1 — Supplementary Information. [file 41598_2022_11099_MOESM1_ESM.docx]

**Supplementary Information (SI) Text**

Analytical modelling of soil porosity and bulk density across the soil organic matter and land-use continuum

D.A. Robinson,^1^* A. Thomas,^1^ S. Reinsch,^1^ I. Lebron,^1^ C. Feeney,^1^ L. Maskell, ^2^ C. Wood, ^2^ F. Seaton, ^2^ B.A. Emmett,^1^ B.J. Cosby^1^

**SI Introduction and framing**

**1.0 Soil formation factors that contribute to soil structure globally**

In the temperate northern latitudes SOM is expected to play the dominate role in structure formation and aggregation, where as in drier Mediterranean climates calcium carbonate will be more prominent, while in highly weathered tropical soils leaching processes and binding agents such as sesquioxides of iron and aluminium will impart a significant imprint on the soils porosity and BD. The important role of SOM in soil structure and impact on processes is increasingly appreciated, but hotly debated ^1^. A unifying model of porosity/BD must however, go beyond aggregation to encompass organic soils and peat in northern latitudes. Any model of porosity/BD developed incorporating SOM will, by the vulnerability of SOM to climate and land use change, be dynamic in nature. National monitoring of SOM reports that in temperate climates changes have occurred over recent decades, especially in cultivated soils where monitoring reports a decline in SOM ^2^. Historical national soil monitoring scarcely made repeat measurements, especially of BD or porosity. Therefore, we don’t have much evidence, and can only speculate that changes in SOM could be leading to changes in soil physical behavior. The inclusion of SOM into models as a variable that helps describe the soil pore space and its changes is therefore important to predict porosity/BD dynamics and determine the scale and magnitude of hydrological and biogeochemical feedbacks resulting from changes to SOM. Thus we consider our work here as part of a broader framework that must develop to describe soil structure in a global context recognizing the importance of soil forming factors in developing future theory describing soil pore space evolution, structure and change in soils.

**SI Theory**

**2.0 Conceptual theory**

The material composition, (i.e. mineral and organic matter) and the microscale characteristics of soil particles, (i.e. shape and size distribution), when acted upon by the forces of nature, arrange themselves in a way that leaves space in between, filled by air or water. The resulting mixtures are characterized by two fundamental emergent bulk properties, the porosity and its reciprocal the BD, both of which are scalars. Study of soil porosity emergence and change therefore demands a stereoscopic view, both macroscopic and microscopic ^3^. A fundamental challenge in environmental science is therefore, determining how climate, land use or management change will impact soil porosity and BD and their ability to regulate processes and store materials on which the planets life support systems rely. We probe the porosity using an analytical framework developed in the following.

**3.0 Theory development for the porosity and bulk density of soils (conservative mixing)**

Determination of the bulk density of a mixture of two materials with different bulk density assumes that the volume remains the same (i.e. conservative mixing).

$\rho_{b}=\frac{M_{T}}{V_{T}}$ S1

Where ρ_b_ is the bulk density of the soil, M_T_ is the total mass of the soil and V_T_ is the total volume of the soil.

$\rho_{b}=\frac{M_{T}}{V_{X}+V_{Y}}$ S2

If the volume of mixing remains the same then we can split the volume into that of two components, V_X_ and V_Y_. Where V_T_ is the sum of V_X_ and V_Y_. But we know that the volume of each component is equal to:

$V_{X}=\frac{M_{X}}{\rho_{bX}}$ S3

If we replace the volumes in Eq S2 with the alternative expression for V_X_, we get:

$\rho_{b}=\frac{M_{T}}{\frac{M_{X}}{\rho_{bX}}+\frac{M_{Y}}{\rho_{bY}}}$ S4

This can now be rearranged:

$\rho_{b}=\frac{M_{T}\rho_{bX}\rho_{bY}}{M_{Y}\rho_{bX}+M_{X}\rho_{bY}}$ S5

$\frac{1}{\rho_{b}}=\frac{M_{Y}\rho_{bX}+M_{X}\rho_{bY}}{M_{T}\rho_{bX}\rho_{bY}}$ S6

$\frac{1}{\rho_{b}}=\frac{M_{X}}{M_{T}}.\frac{1}{\rho_{bX}}+\frac{M_{Y}}{M_{T}}.\frac{1}{\rho_{bY}}$ S7

$\rho_{b}=\frac{1}{\frac{M_{X}}{M_{T}}.\frac{1}{\rho_{bX}}+\frac{M_{Y}}{M_{T}}.\frac{1}{\rho_{bY}}}$ S8

$\frac{M_{X}}{M_{T}}=(X_{X})Mass fraction ofX$ S9

$\rho_{b}=\frac{1}{\frac{X_{X}}{\rho_{bX}}+\frac{X_{Y}}{\rho_{bY}}}$ S10

We can now replace the *X*_x_ with the soil organic matter (SOM) measured for example by loss on ignition (LOI) and *X*_y_ with the soil mineral fraction (1-SOM).

$\rho_{b}=\frac{1}{\frac{SOM}{\rho_{bOM}}+\frac{1-SOM}{\rho_{bM}}}$ S11

Or:

$\frac{1}{\rho_{b}}=\frac{SOM}{\rho_{bOM}}+\frac{1-SOM}{\rho_{bM}}$ S12

Where the bulk density of organic matter (ρ_bOM_) and mineral (ρ_bM_) is that for the ‘pure’ state, limit or end members. In the case of spheres in a maximally jammed random state that is ~1.7 (g cm^-3^) and for a face centered cubic lattice it’s 1.96 g cm^-3^ assuming a particle density of 2.65 g cm^-3^; while for organic matter it is ~0.1 (g cm^-3^). Support for similar values are found in the literature, the Adams-Stewart (AS) model ^4^ was applied to a small data set containing two podsols and proposed values for ρ_OM_ of 0.223 g cm^-3^ and ρ_M_ 1.27 g cm^-3^ for the soils. Rawls ^5^ wanted to predict BD to help estimate soil hydraulic functions and tested the equivalent of Equation S11 for BD on a large USDA data set n=2721, obtaining an average value for ρ_OM_ of 0.224 g cm^-3^; however, the SOM content was limited to the range from 0.1-12.5%. Values for the mineral BD were then estimated based on texture. Hallett ^6^ also tested the AS model on the UK Landis data set n=1568, considering it simply as an empirical model that was outperformed by alternative statistical models, predicting a maximum ρ_M_ 1.8 g cm^-3^ and minimum value ρ_OM_ 0.25 g cm^-3^. Hallett ^6^ noted it performed poorly in organo-mineral and organic soils using a fixed value for ρ_OM_ of 0.224 g cm^-3^. Perie and Ouimet ^7^ studied forest soils with a range of SOM and found that ρ_M_ 1.767 g cm^-3^. While, ^8^ used a slightly different approach and determined that ρ_M_ lay in the range 1.80–2.10 g cm^-3^. Tranter et al. ^9^ also applied the AS model in a thoughtful piece of work aiming to extend our physical understanding of BD. They observed maximum BD’s close to 1.8 and found that adjustment of ρ_OM_ from a value of 0.224 g cm^-3^, for a mostly mineral soil data set, made no improvement to the fit. They made an interesting observation that the Adams-Stewart model had the best r^2^ but the worst RMSE, indicating it captured the trend of the data but that there was a lot of scatter. The realistic trend but broad scatter was also evident in the data presented by Perie and Ouimet ^7^. As statistical methods developed in complexity better predictors have been found ^6, 10^. However, the associated increase in model complexity has led to a rethink by some, captured by Tranter et al ^9^ stating, ‘*It is our experience and strong belief that better models and a better understanding of one’s data result from focused data analysis, guided by substantive theory*’, a theme we develop.

Developing the theory further we know that the bulk density for the soil (Equ S11) is related to the porosity (φ) by the particle density of the soil:

$\varphi=1-\left( \frac{\rho_{b}}{\rho_{s}} \right)$ S13

Both the bulk density of the soil and the particle density of the soil are mixtures that when using mass fractions are averaged according to the harmonic mean (volume according to the arithmetic mean) and should be mixed in the same way. Therefore if $\rho_{ss}$ is the particle density (g cm^-3^) of the soil mixture, then it is also a non-linear mixture of the particle density of the organic particle density (ρ_sOM_) and its complement the mineral particle density (ρ_sM_):

$\rho_{ss}=\frac{1}{\frac{SOM}{\rho_{sOM}}+\frac{1-SOM}{\rho_{sM}}}$ S14

This model is demonstrated to work well in Figure S1 using the data of Ruehlmann ^11^. The porosity for the soil can now be determined given we have equations for the soil bulk density (Equ S11) and the soil particle density (Equ. S14):

$\varphi=1-\left[ \left[ \frac{1}{\frac{SOM}{\rho_{bOM}}+\frac{1-SOM}{\rho_{bM}}} \right]\div\left[ \frac{1}{\frac{SOM}{\rho_{sOM}}+\frac{1-SOM}{\rho_{sM}}} \right] \right]$ S15

Which simplifies to Equ 1a in the main text:

$\varphi=1-\left[ \left[ \frac{SOM}{\rho_{sOM}}+\frac{1-SOM}{\rho_{sM}} \right]\div\left[ \frac{SOM}{\rho_{bOM}}+\frac{1-SOM}{\rho_{bM}} \right] \right]$ S16

It is Equ S16 that forms the basis of further development by linking together the macroscale model with the grain scale modelling of Song et al. ^12^. This results in an equation where the porosity is now a function of the ‘pure’ bulk densities and the particle densities of the materials (Equ 1b and Equ 4b combined from the main text):

$\varphi=1-\left[ \left[ \frac{SOM}{\rho_{sOM}}+\frac{1-SOM}{\rho_{sM}} \right]\div\left[ \frac{SOM}{\frac{\rho_{sOM}z}{z+2\sqrt{3}}}+\frac{1-SOM}{\frac{\rho_{sM}z}{z+2\sqrt{3}}} \right] \right]$ S17

Here we make the assumption that mineral materials maintain a geometric factor for spheres while the geometric factor for organic materials like peat will alter bringing in a geometric term *GF* .

$\varphi=1-\left[ \left[ \frac{SOM}{\rho_{sOM}}+\frac{1-SOM}{\rho_{sM}} \right]\div\left[ \frac{SOM}{\frac{\rho_{sOM}z(\mu_{OM}))}{z(\mu_{OM}))+GF}}+\frac{1-SOM}{\frac{\rho_{sM}z(\mu_{M}))}{z(\mu_{M}))+2\sqrt{3}}} \right] \right]$ S18

Hence, a new model is obtained, describing the porosity as a function of its macro and grain scale components of the soil that should serve as an upper bound for the porosity.

**Table S1.** Median coordination number (CN) predicted when all other parameters held constant such as GF (117); CN used in the model without texture 9.

|  |  |  |  |  |  |
| --- | --- | --- | --- | --- | --- |
|  |  |  | Texture acronym |  | Median CN |
| Clay |  |  | Cl |  | 10.5 |
| Clay loam | |  | ClLo |  | 11.3 |
| Loamy sand | |  | LoSa |  | 9.3 |
| Sand |  |  | Sa |  | 7.3 |
| Sandy clay loam | | | SaClLo |  | 10.6 |
| Sandy loam | |  | SaLo |  | 10.3 |
| Sandy silty loam | | | SaSiLo |  | 10.3 |
| Silty clay | |  | SiCl |  | 10.5 |
| Silty clay loam | | | SiClLo |  | 11.1 |
| Silty loam | |  | SiLo |  | 8.9 |
| Organic |  |  | Organic |  | 9.9 |

**Figure S1** Ruehlmann ^11^ particle density data and Equ S14 fitted.


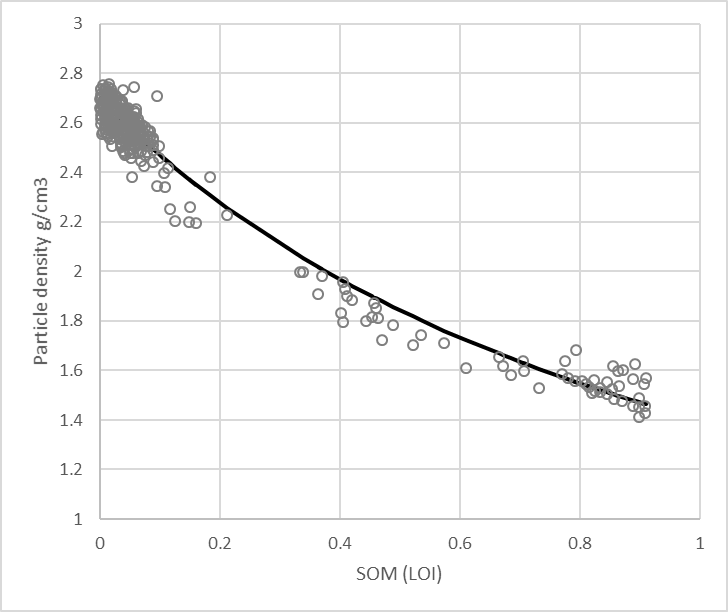


### **Figure S2** The analytical model performance on the bulk density data, equivalent to the porosity data in Figure 1D.


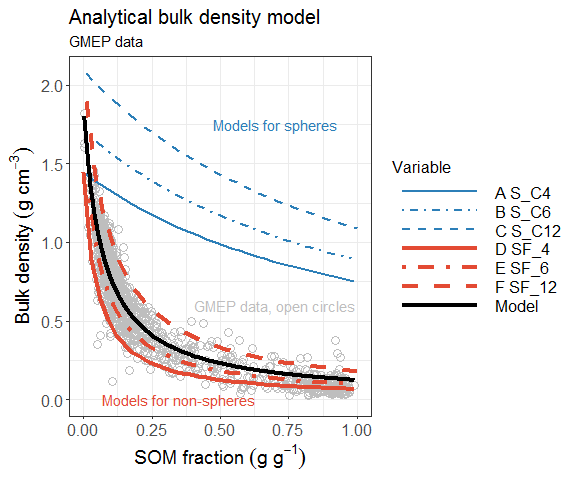


**
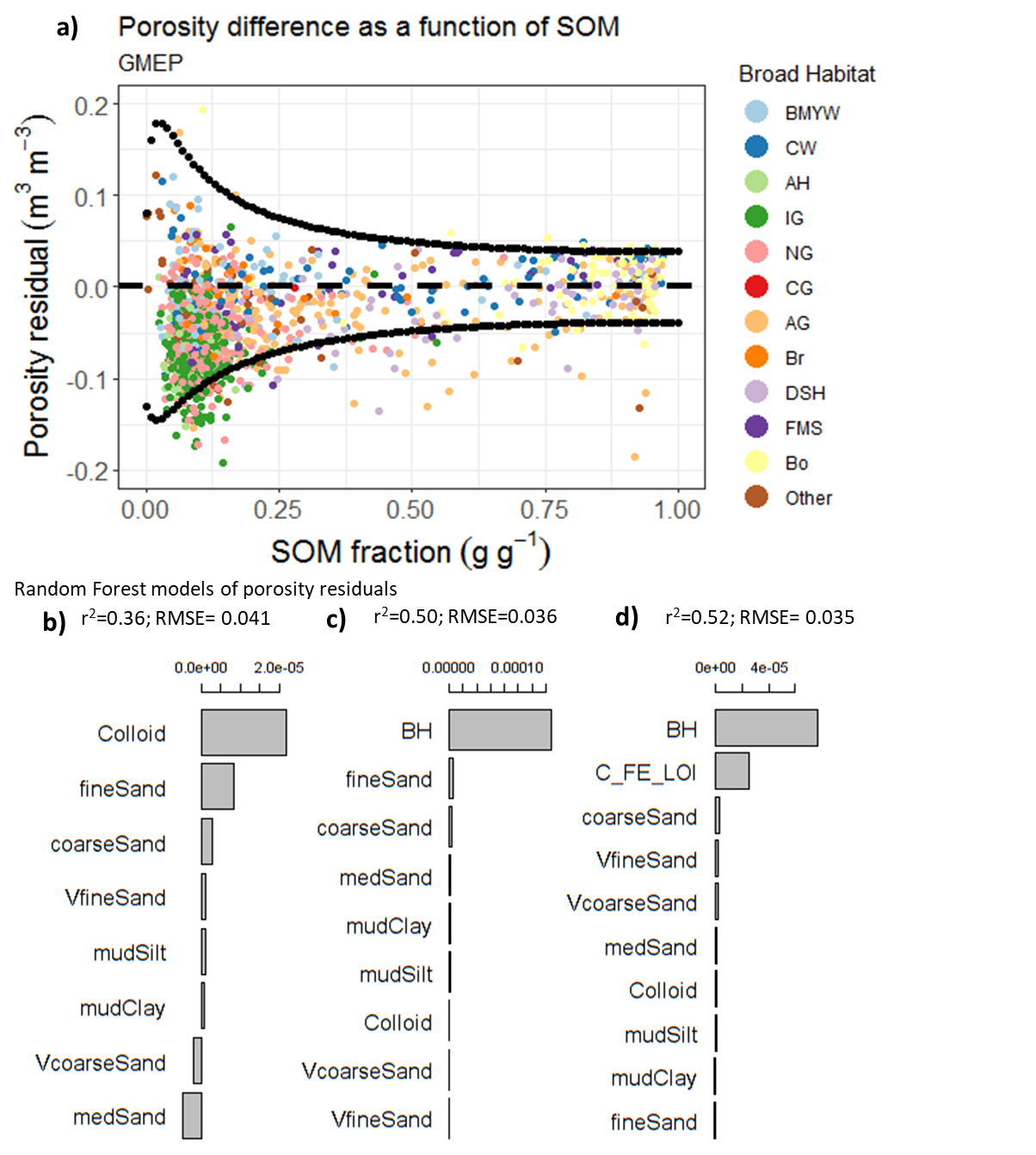
Figure S3** Porosity residuals from the analytical model (Model 3, Table 1) as a function of SOM, where a) displays the raw data behind the means in Figure 2;

### **Figure S4** GMEP histogram of soil organic matter


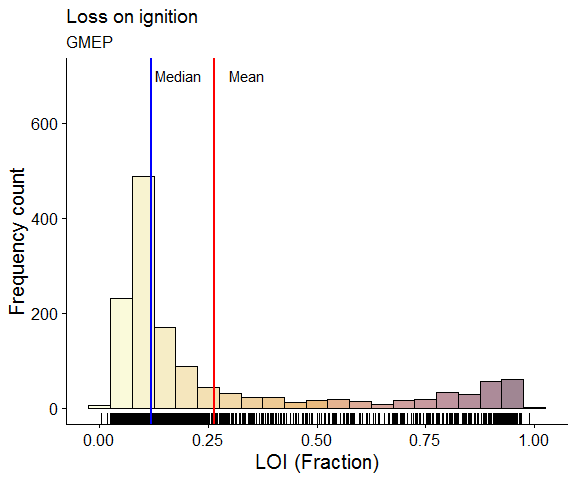


**Figure S5** GMEP histogram of soil bulk density


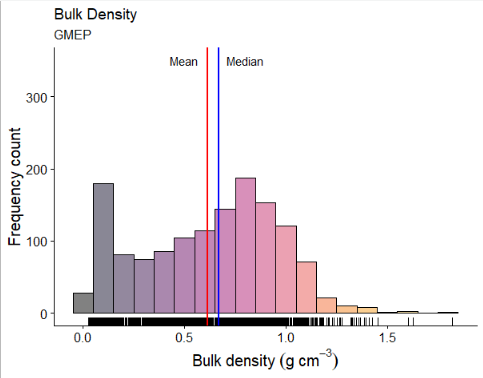


**Figure S6** Countryside Survey histogram of soil organic matter


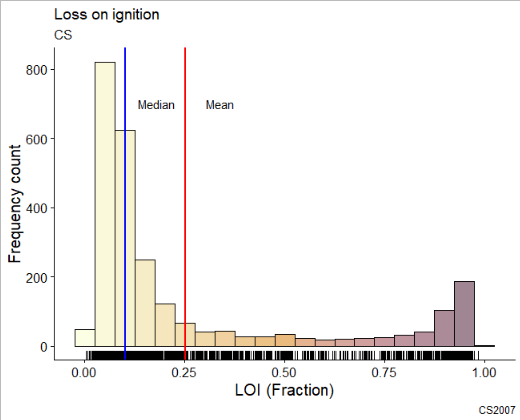


**Figure S7** Countryside Survey histogram of soil bulk density


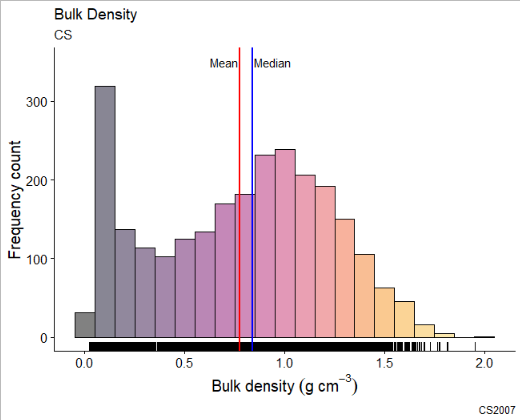


References

1. Kravchenko A, Otten W, Garnier P, Pot V, Baveye PC. Soil aggregates as biogeochemical reactors: Not a way forward in the research on soil–atmosphere exchange of greenhouse gases. *Global change biology* **25**, 2205-2208 (2019).

2. Reynolds B*, et al.* Countryside Survey: National “Soil Change” 1978–2007 for Topsoils in Great Britain—acidity, carbon, and total nitrogen status. *Vadose Zone Journal* **12**, 1-15 (2013).

3. Scarlett B, Van Der Kraan M, Janssen R. Porosity: a parameter with no direction. *Philosophical Transactions of the Royal Society of London Series A: Mathematical, Physical and Engineering Sciences* **356**, 2623-2648 (1998).

4. Adams W. The effect of organic matter on the bulk and true densities of some uncultivated podzolic soils. *Journal of Soil Science* **24**, 10-17 (1973).

5. Rawls WJ. Estimating soil bulk density from particle size analysis and organic matter content1. *Soil Science* **135**, 123-125 (1983).

6. Hallett S, Hollis J, Keay C. Derivation and evaluation of a set of pedogenically-based empirical algorithms for predicting bulk density in British soils.) (1998).

7. Perie C, Ouimet R. Organic carbon, organic matter and bulk density relationships in boreal forest soils. *Canadian journal of soil science* **88**, 315-325 (2008).

8. Ruehlmann J, Körschens M. Calculating the effect of soil organic matter concentration on soil bulk density. *Soil Science Society of America Journal* **73**, 876-885 (2009).

9. Tranter G*, et al.* Building and testing conceptual and empirical models for predicting soil bulk density. *Soil Use and Management* **23**, 437-443 (2007).

10. Ramcharan A, Hengl T, Beaudette D, Wills S. A soil bulk density pedotransfer function based on machine learning: a case study with the NCSS soil characterization database. *Soil Science Society of America Journal* **81**, 1279-1287 (2017).

11. Ruehlmann J. Soil particle density as affected by soil texture and soil organic matter: 1. Partitioning of SOM in conceptional fractions and derivation of a variable SOC to SOM conversion factor. *Geoderma* **375**, 114542 (2020).

12. Song C, Wang P, Makse HA. A phase diagram for jammed matter. *Nature* **453**, 629-632 (2008).
